# Canine organoids: state-of-the-art, translation potential for human medicine and plea for standardization

**DOI:** 10.3389/fvets.2025.1562004

**Published:** 2025-05-09

**Authors:** Kim Verduijn, Hilde de Rooster, Evelyne Meyer, Jonas Steenbrugge

**Affiliations:** ^1^Small Animal Department, Faculty of Veterinary Medicine, Ghent University, Merelbeke, Belgium; ^2^Cancer Research Institute Ghent (CRIG)-Veterinary Oncology Network (VON), Ghent, Belgium; ^3^Laboratory of Biochemistry, Department of Veterinary and Biosciences, Faculty of Veterinary Medicine, Ghent University, Merelbeke, Belgium

**Keywords:** organoid, tumoroid, dog, cross health, culture conditions, methodological rigor, comparative medicine

## Abstract

Organoids have already shown great promise as research tools in human medicine. However, in veterinary medicine, such applications are limited and largely confined to canine organoids. In the Cross Health context, the potential of canine organoids lies in the translation to human diseases, such as cancer. This review provides a state-of-the-art, highlights the current challenges, and at first compares the reported culture conditions of canine organoids derived from both non-neoplastic and neoplastic tissue (i.e., tumoroids), identifying substantial gaps and discrepancies in used culture methods. We make a plea for the standardization of canine organoid culture characteristics and increased rigor in parameter reporting, which will ultimately enhance the reproducibility and applicability of canine organoids in both veterinary and human medicine, especially in the oncology field.

## 1 Introduction

Cell cultures are an essential intermediate step to study the biology and development of disease in an organism (1). These *in vitro* models typically rely on 2D cell lines. Interestingly, the past decade, regenerative medicine studies increasingly reported the use of stem cells to create 3D miniature organs in culture, so called “organoids” (2–6). There are four key stem cell types that can be used to generate organoids: embryonic stem cells (ESCs), induced pluripotent stem cells (iPSCs), adult stem cells (ASCs) and cancer stem cells (CSCs) (1, 2, 7). ESCs are pluripotent and therefore able to differentiate into any desirable body cell type, but their neonatal isolation comes with ethical constraints (8). As a pluripotent alternative, iPSCs rely on a set of transcription factors for their conversion from adult somatic cells such as fibroblasts (8, 9). Both ESCs and iPSCs can generate complex organoids with mesenchymal, epithelial and even endothelial structures, but rarely reach adult tissue stage (2, 8). To this end, multipotent ASCs that differentiate into distinct epithelial cell types for tissue repair and can generate adult tissue organoids, are recommendable when studying adult tissue biology and disease (2, 8). CSCs, a term often used interchangeably with tumor-initiating cells and representing a small subpopulation of (A)SCs that drive tumor growth *in vivo*, can be directly used from tumor resections or biopsies to generate so called “tumoroids” (2).

Overall, organoids better mimic the cell type composition, architecture and, to a certain extent, functionality of differentiated tissues than classical cell lines (1–6). As a result, a superior representation of both healthy and pathological conditions in the living organism is obtained (1–6). The current applications of organoids are fourfold, i.e., (1) studying organogenesis and homeostasis, (2) progressing regenerative medicine, (3) disease modeling, and (4) pharmacological and therapeutic parameter testing (2, 5). These main applications were initially confined to human organoids, which are not readily accessible due to tissue availability and ethical constraints (10, 11). In view of the Cross Health concept, which aims to catalyze the translation of research findings between species, dogs as veterinary patients could serve as an excellent alternative model for human patients and offer a more accessible source of viable cells to establish organoids (12–14). Indeed, companion dogs and humans are susceptible to similar diseases due to their similarities in genetic, anatomical and physiological features, as well as their exposure to comparable environmental and nutritional factors (15–21). They are often also treated similarly as human patients by means of surgical interventions or comparable use of drugs with adjusted dosing based on body weight. These arguments collectively highlight dogs as more relevant translational mammalian models for studying diseases compared to the frequently used rodent models (15–21).

The current review provides an overview of the history and evolution that defined the field of organoid research, and summarizes the reported non-neoplastic and neoplastic tissues from which respectively canine organoids and tumoroids have been derived. It also describes their human translation potential.

To define the culture conditions for each type of canine organoid and tumoroid, and identify similarities as well as discrepancies between reported culture parameters, we performed a meta-analysis of 41 original papers published between 1940 and 2023. Of these papers, 28 and 11 were either organoid- or tumoroid-oriented, respectively, and the 2 remaining papers focussed on both organoids and tumoroids, classifying in total 14 and 7 types of canine tumoroids. Of note, 3 papers report multiple types of organoids or tumoroids.

## 2 Review

### 2.1 Historical evolution of human and veterinary organoid and tumoroid research

**Figure 1:**
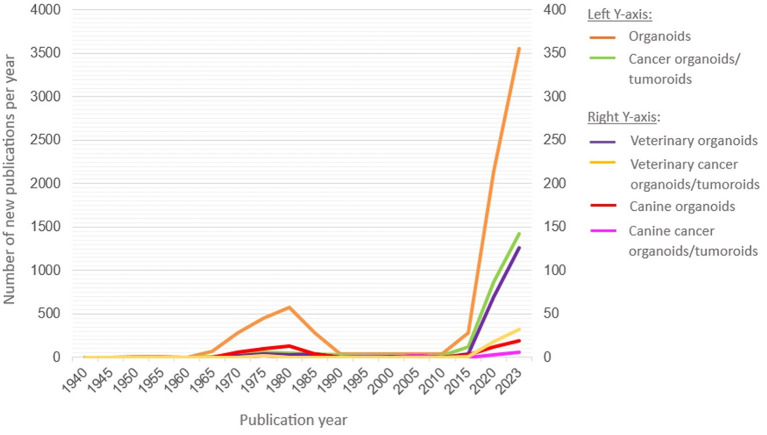
Evolution in time of the number of organoid/tumoroid-oriented publications based on a PubMed search. This literature search includes papers from 1940 till 2023 upon entering the search term “organoids”, “cancer organoids/tumoroids”, “veterinary organoids”, “veterinary cancer organoids/tumoroids”, “canine organoids” or “canine cancer organoids/tumoroids”. The results are presented on two *Y*-axes because of the marked discrepancy in publication numbers between the general (**left axis**), including human, and veterinary (**right axis**) research data and also indicate the associated differences in the application of the organoid vs. tumoroid nomenclature.

Over the past decades, the concept of organoids has gained popularity within human and, albeit to a far lesser extent, in veterinary medicine. Organ regeneration studies served as the foundation for organoid research and can be traced back to as early as 1907 (22–25). The term “organoid” eventually appeared at first in a 1946 case report on cystic organoid teratoma in a 2-month-old infant, and is referred to as “being similar to an organ” (26). The increasing interest in organoid research over the past decades can be illustrated through a literature search in PubMed (Figure 1). An early transient spike in published reports on organoids can be observed between 1965 and 1990, which could be explained by the rising interest in organ development and regeneration (2). Moreover, in 1989, Barcellos-Hoff et al. succeeded in the formation of 3D-structured alveoli by cultivating primary mammary cells on a reconstituted basement membrane, which can be regarded as a precursor of an engineered organoid (27). However, between 1990 and 2010, a drop in the number of organoid-oriented publications occurred, potentially as the result of economic events such as the Fall of the Berlin Wall in 1989, inducing a lack of financial resources for novel research strategies (2). The resumed popularity of organoids since 2010 has been linked to the research groups of Clevers and Sasai, following their high impact publications in 2009 and 2011, respectively, on specific stem cell subtypes (28, 29). These research groups eventually shaped the current definition of organoids. The establishment of the first veterinary organoids in 2009 and subsequent awareness of the numerous benefits of organoids resulted in a rising interest for this research tool, especially in cancer research (Figure 1). Indeed, tumoroids are currently recognized as a superior alternative to earlier cell culture models with a similar architecture as the native tumor and associated extracellular matrix (ECM) (2, 5, 11, 30–32).

### 2.2 Types of canine organoids and tumoroids

Canine organoid cell culture models derived from non-neoplastic tissues are more reported than their neoplastic tissue-derived counterparts (Table 1). Moreover, till date, up to 18 different canine organs/organ systems have been explored for their organoid and/or tumoroid development capacity. Each of these culture models has been linked to a tissue-specific disease in dogs, and can also be translated to humans. In this section, we classify all established canine organoids and tumoroids by tissue type and discuss them in order of importance and chronological order, i.e., based on the year that they were first reported.

**Table 1:** Overview of the reported canine organoids and tumoroids in the literature.

**Tissue type**	**First organoid report**	**First tumoroid report**
Intestine	2009 (30)	/
Endometrium	2009 (31)	/
Mammary gland	2017 (48)	2017 (48)
Oviduct	2023 (54)	/
Liver	2015 (55)	/
Heart	2015 (60)	/
Hippocampus	2015 (65)	/
Spinal cord	2021 (67)	/
Prostate	/	2017 (69)
Skin	2018 (73)	2022 (49)
Bladder	2022 (79)	2019 (80)
Kidney	2019 (85)	/
Pituitary gland	2021 (91)	2021 (91)
Thyroid gland	/	Follicular: 2021 (95) Medullary: 2023 (96)
Lung	2022 (47)	2022 (49)
Pancreas	2022 (47)	/
Cornea	2022 (102)	/
Mesothelium	/	2023 (106)

#### 2.2.1 Intestinal system

Canine intestinal organoids, generally derived from leucine-rich repeat-containing G-protein coupled receptor 5 (LGR5)-expressing ASCs in the epithelial crypts from the canine intestinal tract, were one of the first canine organoid models to be established. More specifically, they were initially used in 2009 by Agopian et al. to observe the normal biology of the gastrointestinal system (12, 30), and have become the most reported canine organoids since then (17, 30, 33–43). They have even proven useful to screen therapeutic efficacy against multiple human gastrointestinal diseases (12, 17, 33, 44). Yet, tumoroid cultures from canine intestinal carcinomas have not been thoroughly described in the literature.

#### 2.2.2 Female reproductive system

The female reproductive system comprises the ovaries, oviducts, uterus, vagina and mammary glands (45). Currently, there are no reports on organoids from canine ovarian or vaginal tissue. Canine endometrial organoids derived from ASCs were first reported in 2009 by Stadler et al., and provide an important model for endometrial diseases in both dogs (e.g., cystic endometrial hyperplasia and pyometra) and humans (e.g., endometriosis, a chronic disease in which the inner uterus lining grows outside the uterus) (31, 32, 46, 47).

In 2017, Cocola et al. established both canine mammary organoids from ASCs and tumoroids from CSCs as a model for *in vitro* drug testing and identifying specific tumor mutations that can predict therapeutic outcome (48). Canine mammary tumoroids have become one of the most often generated tumoroid types (11, 48–50), partly due to their frequent diagnosis in female dogs, accounting for almost 50% of all cancers and >80% of all reproductive tumors (48, 51, 52). In comparison, human breast cancer is the most frequently occurring and deadly cancer in women worldwide, accounting for 25% of all human cancers (48). Indeed, given their similarities regarding age at onset, predominance of carcinomas, environmental risk factors, histological features, prognostic factors, metastatic pattern, and molecular and genetic characteristics, canine mammary tumoroids serve as a translational model for human breast cancer (48, 50, 51, 53).

Canine oviductal epithelial organoids from oviductal PAX8^+^ ASCs were only recently established in 2023 by Lawson et al. to investigate fertilization and oviductal pathologies (54). However, the canine-specific physiology of the oviductal tissue limits the translational potential of these organoids to other species (54). The authors even showed that porcine, equine and bovine oviductal organoids better resembled human oviducts than canine oviductal epithelial organoids (54). Nevertheless, these latter organoids can still be very useful in studying oviductal biology in canine veterinary medicine, potentially providing insight into evolutionary conserved mechanisms that also occur in humans.

#### 2.2.3 Hepatobiliary system

Canine hepatic organoids established from ASCs in the liver can be used to study multiple liver pathologies, including copper storage disease and portosystemic shunts (37, 47, 55–58). The first canine hepatic organoids were established in 2015 by Nantasanti et al. from dogs with copper metabolism domain containing 1 (COMMD1) deficiency, a genetic defect that results in hepatic copper accumulation (55). More recent studies in 2019 and 2020 by Wu et al. and Kruitwagen et al., respectively, used this model for translational purposes and studying copper storage disease in humans (57, 58). In 2017, Van den Bossche et al. established hepatic organoids from dogs with portosystemic shunts to further understand hepatic lipid accumulation and provide insight into non-alcoholic fatty liver disease, one of the most common human liver disorders (56). Tumoroids of the hepatobiliary system have not been reported yet in the literature (59).

#### 2.2.4 Cardiovascular system

The first canine cardiac organoids have been established in 2015 by Hensley et al. through 3D culturing of ASCs (referred to as cardiosphere-derived cells), creating a heterogenous population of cardiac cells with canine and human healthy heart tissue resemblance (60). Cardiac diseases occur frequently in both human and veterinary medicine (60–63), and dogs are widely used as experimental animal models for the human cardiovascular system (64). Although translational experiments cannot solely rely on canine model systems due to anatomical differences in for example the coronary circulation, canine cardiac organoids can still provide a potential solution to the availability constraints of human heart-forming organoid models (60, 64).

#### 2.2.5 Nervous system

Cerebral organoids are mostly created from e.g., primate species, although their establishment is hampered by ethical constraints (65). Yet, there is potential for translational neuroscience in dogs, given that the canine brain, and more specifically the cortex and frontal lobe, is comparable to the human brain in its basic anatomy (66). Moreover, it has been verified that dogs have memory, experience emotions and could suffer from similar mental illnesses as their human counterparts (66). Canine neurological organoids have currently only been derived from the hippocampus in 2015 by Lowe et al. through isolation of adult neural stem cells, and from the spinal cord in 2021 by Santos et al. through isolation of embryonic neural stem cells from late stage canine fetuses, providing more accessible alternatives to primate cerebral organoids (65, 67).

#### 2.2.6 Male reproductive system

The canine male reproductive system comprises the scrotum, testes, epididymis, deferent ducts, spermatic cords, prostate, penis and urethra (68). There have been no reports so far on canine organoids derived from any part of the healthy male reproductive system. In contrast, canine prostate tumoroids, derived from prostate CSCs that are excreted in urine, were first established in 2017 by Usui et al., and allowed to select the most effective treatment for a specific patient (69). Strikingly, dogs are the only other large mammalian species that can develop spontaneous prostate tumors with similar cellular and stromal constituents as human prostate tumors (69–71). Yet, canine prostate adenocarcinoma are mostly classified as highly invasive and metastatic, whereas prostate tumors in humans are often low grade and slowly progressive (70–72).

#### 2.2.7 Skin

In 2018, Wiener et al. established the first canine keratinocyte organoids from adult hair follicle stem cells, developing a dermatological organoid system for studying non-neoplastic skin disorders (73). In 2021, the organoid culture model was further optimized by the same group (74). Dermatological disorders, such as alopecia, commonly occur in both dogs and humans (73, 74). However, as dermatological organoid systems lack connective tissues, blood vessels and immune cells that are present in normal skin, further optimization is required to allow their clinical application (73–75).

Despite the many types of skin tumors that occur in dogs (76), canine melanoma tumoroids are currently the only type of skin tumoroids that have been established. More specifically, Abugomaa et al. reported their generation from CSCs in 2022, albeit without adding ECM components to the culture media (referred to as a 2.5D organoid culture model) (49). Melanoma is a very aggressive and metastatic tumor that occurs in both dogs and humans (77). Although melanoma presents as a skin cancer in 80% of human patients, it mostly occurs in the oral cavity of canine patients. Of note, melanocytes cannot be solely classified as skin cells, since they also occur in many other locations such as the mouth and lips, inner ear, nervous system, heart, eye and nails (76–78).

#### 2.2.8 Urinary system

Canine bladder organoids were first established from adult bladder basal cells with high stemness in 2022 by Elbadawy et al., shortly followed by Zdyrski et al. (47, 79). In both studies, these organoids served as a “healthy” model system to unravel the mechanisms underlying the neoplastic transformation of the bladder tissue and to identify novel biomarkers in bladder cancer (47, 79). Canine bladder tumoroids were developed earlier than their healthy counterparts, and more specifically in 2019 by Elbadawy et al., as an important model to determine suitable chemotherapy in canine and human patients (80). Besides mammary tumoroids, CSC-derived bladder tumoroids are one of the most reported canine tumoroids (49, 80–83). Bladder cancer is the most common type of neoplasia of the urinary tract in both dogs and humans, comprising 1–2% of all naturally occurring tumors in dogs (79–84). Canine bladder cancer has a poor prognosis and is often diagnosed at a late stage. Consequently, 90% of canine bladder cancer cases are classified as intermediate or high grade muscle invasive urothelial carcinoma, also known as transitional cell carcinoma, strikingly resembling human bladder cancers based on aggressiveness and cellular components (79–84).

The establishment of canine renal organoids from adult kidney epithelial stem cells in 2019 and 2022 by Chen et al. and Zdyrski et al., respectively, was reported as an opportunity to accelerate drug screening and explore applications for regenerative medicine, ultimately providing improved outcome for renal disorders in both dogs and humans (47, 85). To this end, canine renal organoids can become important models for chronic kidney disease, which is suboptimally treated in both species (85, 86). Indeed, treatment for end-stage renal disease in humans solely relies on either kidney transplantation or hemodialysis for long-term survival. In dogs, both treatment options are rarely performed due to ethical constraints and a high morbidity and mortality rate associated with the procedures (86–89).

#### 2.2.9 Endocrine system

The endocrine system consists of multiple tissues, including the pituitary and thyroid gland, that release hormones in the circulation (90). In 2021, although only reported as an abstract that was published in conference proceedings, Sanders et al. established both pituitary organoids and tumoroids to study Cushing's disease progression as observed in humans and to identify novel therapeutic targets (91). Although not explicitly stated, ASCs and CSCs, respectively, displaying expression of SOX2 and SOX9 as general stem cell markers, were likely the starting stem cell type for establishment of these canine pituitary organoids and tumoroids. Cushing's disease, associated with hypercortisolism and multiple co-morbidities, is frequently caused by either adrenal or pituitary adenomas (92, 93). Its prevalence is a thousandfold higher in dogs than in humans, suggesting that a large source of canine samples is available to establish canine tumoroids as translational models (91, 94).

Thyroid cancer accounts for 1–2% of all neoplasms in dogs, with 90% being malignant (95, 96). Compared to humans, canine thyroid cancer has a higher incidence of lung metastasis and is not associated with sex predisposition (97). From all canine thyroid carcinomas, 70% can be classified as follicular and 30% as medullary thyroid carcinoma (95, 96). Although medullary thyroid carcinoma is less invasive and metastatic disease occurs at a slower rate, the prognosis after thyroidectomy is similar for both medullary and follicular thyroid carcinoma (95, 96). The first CSC-derived canine follicular and medullary thyroid carcinoma tumoroids were established in 2021 and 2023, respectively, by Jankovic et al. and Scheemaeker et al., with both studies intending to test therapeutics against thyroid cancer that could benefit dogs and humans (95, 96).

#### 2.2.10 Lung

Canine pulmonary organoids have been described as a suitable model system for human respiratory disorders such as asthma, bronchitis, pneumonia, pulmonary fibrosis, chronic obstructive pulmonary disease and lung cancer (47, 49, 98–100). The first canine pulmonary organoids were established in 2022 and 2023 by Zdyrski et al. and Sato et al., respectively, and relied on the culturing of lung-derived ASCs that matured into alveolar type-2 cells and bronchial epithelial cells (47, 100). Also in 2022 and 2023, Abugomaa et al. and Sato et al., respectively, described the first canine pulmonary tumoroids from CSCs as a model for lung adenocarcinoma (49, 100).

#### 2.2.11 Pancreas

Derived from ASCs in both the endocrine and exocrine parts of the pancreas, canine pancreatic organoids have been established in 2022 by Zdyrski et al. as alternative model to human pancreatic organoids, and to study diabetes and pancreatitis, the most common diseases of the canine exocrine pancreas (47, 101). Canine pancreatic tumoroids as a model for lethal pancreatic cancer in humans have not yet been reported.

#### 2.2.12 Cornea

Canine organoids derived from the adult limbal epithelial stem cells of the cornea, with the typical corneal markers including transformation-related protein 63 (p63) maintained upon culturing, were first reported in 2022 by Bedos et al. to reliably study corneal pathology such as corneal blindness (61, 102, 103). This group of eye disorders that progressively affects the transparency of the cornea, is mostly observed in dogs as a result of chronic superficial keratitis and keratoconjunctivitis sicca (104). In humans, it is mostly caused by infectious disease and predisposing factors, such as the use of contact lenses and steroids (103).

#### 2.2.13 Mesothelium

The mesothelium consists of a monolayer of cells that line the serous cavities, providing a protective surface for the internal organs (105). Currently, there have been no reports on canine organoids from healthy mesothelium. In contrast, canine malignant mesothelioma tumoroids derived from CSCs in pleural effusion were at first established in 2023 by Sato et al. to develop novel treatment options for both dogs and humans (106). Malignant mesothelioma is a rare, aggressive and drug-resistant tumor with similar characteristics in both species (7, 106). However, whereas human malignant mesothelioma is mostly localized in the mesothelial lining of the pleura, canine malignant mesothelioma may also arise from the peritoneum or pericardium (7, 106).

### 2.3 Culture conditions for canine organoids and tumoroids

The procedure to establish organoids and tumoroids comprises 3 steps, i.e., (1) cell isolation, (2) cell seeding and (3) cell culture (107). To establish a state-of-the-art methodological overview, we analyzed 41 original papers on canine organoids/tumoroids, identifying similarities and differences, as well as significant gaps, in parameter reporting (Supplementary Tables S1, S2).

#### 2.3.1 Cell isolation

The successful generation of organoids and tumoroids highly depends on the starting stem cell type, i.e., ESCs, iPSCs, ASCs or CSCs (1, 2, 108). ASCs and CSCs are most frequently used for the formation of emerging canine organoids and tumoroids, respectively, and are well-documented in the analyzed literature (89% and 94% of canine organoid and tumoroid papers, respectively).

Importantly, the characteristics of these starting stem cell types are typically donor-dependent, influenced by the strain, age, and sex of the dog as well as the location of their retrieval (109, 110). Although the tissue retrieval location is described in detail in most publications, we observed that, in contrast to the tumoroid papers, the majority of the analyzed organoid papers did not consistently report on the other crucial host parameters (i.e., 64% and 19% of canine organoid and tumoroid papers, respectively) (Supplementary Tables S1, S2).

To prepare organoids/tumoroids from a single cell suspension, the desired starting stem cell type needs to be isolated, most commonly from surgery-derived (64% and 56% of canine organoid and tumoroid papers, respectively) and/or necropsy-derived tissue (67% of canine organoid papers) (74). The dissected tissue is then rinsed to remove contaminants and minced to increase the total surface area (111). Several cell wash steps (83% and 69% of canine organoid and tumoroid papers, respectively, with 67% of organoid and 25% of tumoroid papers mentioning the number of wash steps) and enzymatic digestion (42% and 50% of canine organoid and tumoroid papers, respectively) are first performed on the isolated tissue to obtain a stem cell pool (111–113). More specifically, cells are washed with a physiological buffer, such as phosphate buffered saline, to remove metabolic waste, cell debris and other unwanted components, thereby minimizing risk of overgrowth and purifying the sample for downstream analysis (112, 113). The desired starting stem cells are then isolated using a cocktail of proteolytic enzymes, such as collagenase, dispase, hyaluronidase, trypsin and AccuMax™, to break down the ECM (111, 114). Collagenase, reported in 33% and 50% of canine organoid and tumoroid papers, respectively, specifically cleaves the peptide bonds in collagen (111). Dispase, reported in 17% of canine organoid papers, has a high specificity for collagen IV and fibronectin, and cleaves attachments between cells and the ECM (111). Hyaluronidase, reported in 8% and 13% of canine organoid and tumoroid papers, respectively, cleaves the β1,4-glycosidic bonds in hyaluronan, a structural proteoglycan in the ECM (111). Trypsin or TrypLE™ Express, reported in two neurological organoid papers, a paper on canine mammary tumoroids and a paper on canine transitional cell carcinoma tumoroids, cleaves cell-cell junctions (49, 50, 65, 111). A single study by Lawson et al. (2023), focussing on canine oviductal epithelial organoids, describes the use of AccuMax™ (54). This commercially available enzyme cocktail with both proteolytic and deoxyribonuclease activity can serve as a replacement for collagenase or trypsin (54, 114). Compared to other enzymes, AccuMax™ does not contain mammalian- or bacterial-derived products, resulting in a higher cell culture reproducibility (114). AccuMax™ is very similar to Accutase™, containing the same enzymes at a higher concentration (111, 114).

#### 2.3.2 Cell seeding

Successful cell seeding, depending on technical parameters such as the use of specific tubes/plates and the cell seeding density, highly influences the survival rate and reproducibility of the cell culture (115). Despite their importance, 11% of canine organoid papers fail to report the used tube/tube type for their cultures, and even 47% and 81% of canine organoid and tumoroid papers, respectively, do not report the cell seeding density (Supplementary Tables S1, S2).

#### 2.3.3 Cell culture

To establish a 3D cell culture, specialized culture components and characteristics are required to enhance cell viability and proliferation, including the re-incorporation of a controlled ECM, the use of a specific culture medium and a stable pH (1, 2, 24, 116, 117). The pH of cell cultures is set between 7.2 and 7.4, and is highly influenced by the CO_2_ concentration of the incubator (reported in 67% and 13% of canine organoid and tumoroid papers, respectively) (116–118). The culture medium serves as a source for nutrients and it maintains the correct osmolality (116). It is composed of a basal medium and a number of supplements, both depending on the type of organoid or tumoroid. In the following paragraphs, the cell culture components of the different reported canine organoids (Supplementary Table S1) and tumoroids (Supplementary Table S2) are discussed and compared.

##### 2.3.3.1 Extracellular matrix

Although inclusion of an ECM in the organoid or tumoroid cell culture is fundamental to mimic *in vivo* conditions, 8% and 31% of canine organoid and tumoroid papers, respectively, do not report its use in experiments (2, 24, 119). To enhance reproducibility, a controlled ECM can be provided using either Matrigel^®^ or Basement Membrane Extract (BME/Geltrex™) (2, 24, 119, 120). These matrices are both derived from the natural ECM produced by Engelbreth-Holm-Swarm tumors in mice, which spontaneously develop and are classified as poorly differentiated chondrosarcomas (2, 24, 119, 120). Compared to BME, Matrigel^®^ has a higher level of functional protein and is therefore more frequently used, i.e., in 83% and 50% of canine organoid and tumoroid papers, respectively (119). However, the use of animal-derived ECM in cell culture poses a risk for immunogen and pathogen transfer, limiting the use of organoids and tumoroids for clinical applications. Moreover, due to their animal origin, the use of these matrices also means failing to support the 3R-concept (i.e., the replacement, refinement and reduction of animal models) (2, 10, 121, 122). Although natural, synthetic or protein-engineered hydrogels have already been developed, their use has not reached its full potential (10, 120, 121). Challenges remain as working with these hydrogels requires considerable experience and time due to the need for finetuning (120, 121).

##### 2.3.3.2 Basal culture media

Several basal media variants are used for canine organoid and tumoroid culturing, and were found to be always reported in the analyzed papers (Supplementary Tables S1, S2).

Minimal Essential Medium (MEM), originally developed by Eagle in 1959, is one of the most basic commercial media as it only contains vitamins, essential amino acids, glutamine and inorganic salts (116, 123, 124). Dulbecco's Modified Eagle's Medium (DMEM) is a first variant of MEM and was developed by Dulbecco and Freeman in 1959 (116, 123, 125). It contains fourfold higher concentrations of vitamins and amino acids than MEM, and is widely used in 2D mammalian cell cultures (116, 123). However, as DMEM does not contain proteins, lipids or growth factors, it still requires supplementation of serum (116, 123). Only 2 organoid papers report the use of DMEM as a suitable basal medium (30, 31). Iscove's Modified Dulbecco's Medium (IMDM), first reported in 1978 by Iscove and Melchers, is a variant of DMEM, containing more amino acids, vitamins and inorganic salts (116, 123, 126). IMDM is mainly used for high density cell cultures such as cardiac organoids (116, 123). DMEM/F-12, first reported in 1979 by Barnes and Sato, is a second variant of DMEM that contains the advantages of higher zinc sulfate, putrescine and linoleic acid concentrations as found in Ham's F-12 basal medium (123, 127). Yet, the Advanced DMEM/F-12 formulation requires even 50–90% less serum supplementation, which makes it more suitable for highly sensitive canine organoids and tumoroids, and explains its overall common use (128). More specifically, advanced DMEM/F-12 is reported in 78% and 88% of the investigated canine organoid and tumoroid papers, respectively. Modified Molecular, Cellular and Development Biology (MCDB) 153 medium, developed in 1980 by Peehl et al., is a specialized basal medium that is specifically used for culturing human renal organoids (123). In 2019, Chen et al. also reported its use for canine renal organoid culturing (85). However, in 2022, Zdyrski et al. managed to successfully develop renal organoids by using advanced DMEM/F-12 as alternative basal medium (47). Neurobasal medium, such as NeuroCult™ Neural Stem Cell (NSC) basal medium, was originally formulated by Brewer et al. in 1993 and is specifically used for culturing of central nervous system-derived neural stem and progenitor cells (129, 130). NeuroCult™ is reported in 1 paper focussing on canine organoids derived from the hippocampus (65). Yet, a study on canine spinal cord organoids reported the use of DMEM/F-12 as alternative basal medium (67).

##### 2.3.3.3 Culture media supplements

The number and type of supplements that are provided to the selected basal media depend on the type of organoid or tumoroid that needs to be established (116, 123). More than 10 types of culture media supplements are used for organoid and tumoroid cultures, which will be discussed in alphabetical order in the following paragraphs (Supplementary Tables S1, S2).

Activators of specific pathways are rarely used in currently reported canine organoid and tumoroid cultures. Forskolin, a natural adenylyl cyclase/cyclic adenosine monophosphate (cAMP) signaling pathway activator with potential neuroprotective properties, is only reported in 2 papers focussing on canine keratinocyte organoids and in 1 paper focussing on canine medullary thyroid carcinoma tumoroids (73, 74, 96, 131). Dibutyryl-cAMP activates cAMP-dependent protein kinases and is only reported in 1 tumoroid study (96, 132). Heparin, which is a glycosaminoglycan, has a high protein affinity and enhances cellular proliferation by regulating the activity of growth factors such as Wingless and Int-1 (Wnt) and fibroblast growth factor (FGF) (133). It is reported in 3 papers focussing on canine hippocampus organoids, canine keratinocyte organoids, and both canine mammary organoids and tumoroids (48, 65, 73).

Amino acids, both essential and non-essential, are commonly supplemented to cell cultures as most cell types are unable to produce sufficient amounts of these basic protein building blocks (116, 134). Indeed, the use of L-glutamine or GlutaMAX™, a stabilized alternative, is reported in 69% and 88% of canine organoid and tumoroid papers, respectively (123). N-acetylcysteine (NAC), a supplement form of cysteine, is commonly used as a fast-acting water-soluble antioxidant and is reported in 83% and 94% of canine organoid and tumoroid papers, respectively (135). The addition of both these amino acid supplements in the basal medium is reported in 67% and 81% of canine organoid and tumoroid papers, respectively.

Antibiotic/-mycotic supplementation to the culture medium is important as nutrient-rich cell cultures are prone to fungal and bacterial contamination (116). Still, 22% of the canine organoid papers do not report the use of antibiotics/-mycotics. Our literature search identified reporting of five antibiotics/-mycotics in canine organoid and tumoroid cultures, including penicillin/streptomycin (PS), amphotericin B, trimethoprim sulfamethoxazole (TMS), primocin and nystatin. There is no clear preference for certain antibiotics/-mycotics in the organoid papers, although the use of nystatin has only been reported in 1 paper focussing on canine endometrial organoids (31). In contrast, the tumoroid papers prefer the use of PS and primocin (reported in 81% and 50% of canine tumoroid papers, respectively).

Buffers are necessary to maintain the pH of the cell culture, and are reported in 72% and 94% of canine organoid and tumoroid papers, respectively (116, 117, 123). The studies that do not additionally buffer the basal culture medium potentially rely on the buffering capacity of NaHCO_3_ present in most basal culture media (117). Two different buffers have been used for canine organoid and tumoroid culturing: 4-(2-hydroxyethyl)-1-piperazineethanesulfonic acid (HEPES) and Hank's balanced salt solution (HBSS), with HEPES having an enhanced buffering capacity compared to bicarbonate-based HBSS (136). Consequently, 69% and 94% of canine organoid and tumoroid papers, respectively, report the use of HEPES in cell culture media. HBSS is only described in 2 papers focussing on canine intestinal organoids and canine mammary tumoroids (11, 30).

Growth factors stimulate proliferation, migration or differentiation of cell types. However, as most growth factors are animal-derived, they are posing a risk factor for contamination (123). Epidermal growth factor (EGF) is most commonly used in 3D culture media and reported in 92% of canine organoid as well as all canine tumoroid papers. Heregulinβ-1 (HRGβ-1) and neuregulin-1 (NRG-1) are both members of the EGF family and play a role in the neoplastic transformation of the mammary gland. Consequently, both growth factors are reported in papers focussing on canine mammary tumoroids (11, 50, 137, 138). FGFs represent a large growth factor family, including FGF-1,−2,−7,−10,−18 and −19, and are reported in 42% and 31% of canine organoid and tumoroid papers, respectively. However, canine organoid and tumoroid papers significantly differ in the used FGFs. Insulin-like growth factor-1 (IGF-1) is part of the IGF-family and is only reported in 3 papers focussing on canine intestinal organoids and 1 paper focussing on canine mesothelioma tumoroids (35, 40, 42, 106). Hepatocyte growth factor (HGF) is a more specific growth factor, often used for organoids and tumoroids associated with the digestive system. More specifically, HGF has been reported in 3 papers focussing on canine intestinal organoids and 4 papers focussing on canine hepatic organoids (35, 40, 42, 55–58). Bone morphogenetic protein-7 (BMP-7) is a member of the transforming growth factor-β (TGF-β) superfamily (139) and is reported in 2 papers focussing on canine hepatic organoids (57, 58). Protein factors Wnt3a, R-spondin (Rspo)-1/-3 and Noggin are necessary to generate organoids and tumoroids from ASCs and CSCs, respectively (140). They are often combined (reported in 78% and 38% of canine organoid and tumoroid papers, respectively) either through supplementation of recombinant proteins or through a conditioned medium from other cell lines that are engineered to secrete these protein factors.

Hormones also strongly impact cell growth, proliferation, differentiation and function (116, 123). Gastrin is the most commonly supplemented hormone in 3D culture media and is reported in 64% of canine organoid papers. In marked contrast, it is only reported in 1 paper focussing on canine transitional cell carcinoma tumoroids (83). Other hormones, including hydrocortisone, dexamethasone, insulin, triiodothyronine (T3) and thyrotropin (TSH) are rarely reported in the literature. Hydrocortisone is used to improve the cloning efficiency of glial cells and fibroblasts (123), and is only reported in 2 papers focussing on canine renal organoids and on both canine mammary organoids and tumoroids (48, 85, 116, 123). Dexamethasone, a Notch inhibitor that is used to differentiate ASCs into hepatocytes with acquirement of the correct phenotype and also lowers the risk of cellular apoptosis, is reported in 2 out of 6 studies focussing on canine hepatic organoids (57, 58, 141, 142). Insulin can be used for cell proliferation of many cell types, but requires zinc supplementation to fulfill its proliferative activity and is unstable at 37°C (123). These limitations potentially explain why insulin is only reported in 3 papers focussing on canine renal organoids, canine medullary thyroid carcinoma tumoroids, and both canine mammary organoids and tumoroids (48, 85, 96). T3 is used to specifically enhance kidney and pulmonary epithelial cell proliferation, but is only reported in 1 paper focussing on canine renal organoids and is not mentioned in canine pulmonary organoid papers (47, 85, 100). TSH is important for thyroid cell proliferation, plays a role in the neoplastic transformation and progression of thyroid cancer, and is reported in 1 paper focussing on canine medullary thyroid carcinoma tumoroids (96, 143).

Inhibitors of specific metabolic pathways are commonly used in cell culture media (116). The TGF-β inhibitor A83-01 is the most often supplemented inhibitor for organoid/tumoroid culture expansion, reported in 78% and 88% of canine organoid and tumoroid papers, respectively (144). Rho-associated protein kinase inhibitor Y-27632 enhances the survival of stem cells and is reported in 72% and 44% of canine organoid and tumoroid papers, respectively (145). SB202190, an inhibitor of P38 mitogen-activated protein kinase, inhibits cellular apoptosis, stimulates cellular proliferation and migration in 3D culture models, and is reported in 47% and 31% of canine organoid and tumoroid papers, respectively (146). CHIR99021, an aminopyrimidine derivative, acts as an inhibitor of the enzyme glycogen synthase kinase (GSK) 3 that regulates differentiation of stem cells by impacting iron metabolism (147). Although CHIR99021 is reported in 42% of the canine organoid papers, it is only mentioned in 1 tumoroid paper focussing on canine transitional cell carcinoma tumoroids (83). N-[N-(3,5-difluorophen-acetyl)-l-alanyl]-S-phenylglycine t-butyl ester (DAPT) is a y-secretase inhibitor that, similarly as dexamethasone, is used to acquire the hepatocyte phenotype in cell cultures, and it is reported parallel with dexamethasone in 2 papers focussing on canine hepatic organoids (57, 58, 141, 142).

Multi-component supplements are composed of several factors that generally contribute to cellular growth and viability in 3D culture models. B-27, consisting of 20 different components, is widely used in cell culture media and more specifically reported in 81% and 50% of canine organoid and tumoroid papers respectively (129). N2, consisting of 5 different components, is reported in 58% of canine organoid papers and 1 paper on canine transitional cell carcinoma tumoroids (83, 129). Bovine Pituitary Extract (BPE) contains a variety of growth factors and hormones with antioxidant activity and is reported in 1 paper focussing on canine renal organoids (85, 148). NeuroCult™ Proliferation Supplement is a standardized commercial multi-component supplement for culturing of neural stem cells and is reported in 1 paper focussing on canine hippocampus organoids (65, 149).

Saccharides and their metabolites serve as an energy source in cell culture media (116, 150). Sodium pyruvate, a glucose metabolite, is frequently included in basal media and is therefore not separately reported as supplement in canine organoid and tumoroid papers (151). However, D-sorbitol, a reduced D-glucose, has been reported as supplement in culture media of canine intestinal organoids (30). Of note, 39% of canine organoid papers also report the use of D-sorbitol to maintain tissue viability during washing steps and stem cell isolation prior to organoid culturing.

Serum is one of the main components required to enhance cellular growth and proliferation, and is a source of amino acids, carrier proteins, growth factors, hormones, inorganic salts, lipids, trace elements and vitamins (116, 123). The most widely used type of serum is fetal bovine serum (FBS), which is reported in 75% and 50% of canine organoid and tumoroid papers, respectively. Although serum contains a variety of growth-stimulating and cell-stabilizing factors, it also has a highly variable lot-to-lot composition, which can lead to different research outcomes (123). Moreover, as serum is an animal-derived product, the use of serum-reduced or serum-free media is gaining popularity (123, 152). Alternatively, serum replacement (SR) can be used as a synthetic serum substitute to support cellular growth, but is currently reported in only 1 paper focussing on both canine mammary organoids and tumoroids (48, 123, 152).

Vitamins are typically already included in the basal medium, but some specific vitamins often need to be supplemented due to their limited available amount (116, 123). Nicotinamide, a vitamin B3 derivative that stimulates stem cell differentiation and survival, is reported in 69% and 81% of canine organoid and tumoroid papers, respectively (153). One paper focussing on canine renal organoids also describes the supplementation of L-ascorbic acid 2-phosphate (Asc-2P), a vitamin C derivate, in the cell culture medium (85).

## 3 Discussion

Organoids are 3D *in vitro* models that phenocopy the complex characteristics of corresponding *in vivo* tissues to a much better extent than 2D cultures (1–6). Their uprise is most noticeable in the field of oncology, where they are referred to as tumoroids, accounting for 40% of all organoid studies in 2023 (Figure 1). Indeed, the spontaneous occurrence of canine cancers, sometimes rare in humans, render dogs an accessible source of tumoroids. Canine organoids have also been increasingly implemented for translational research as these can overcome the constraints in the availability of necessary human material and its accompanying ethical issues (10–21). Yet, the establishment of canine organoids also comes with ethical considerations. The sourcing of the tissue from which organoids/tumoroids are eventually developed, should be performed with consent of the pet's owner, in accordance with the ARRIVE guidelines and after approval of the Institutional Animal Care and Use Committee (IACUC). These considerations are in place for tissue harvesting from both live animals, that undergo surgery, and euthanized animals (154). Most importantly, the tissue harvesting should also be performed without impacting animal welfare and should not bring additional harm to the canine patient. Residual material of tissue biopsies or curative surgical resections that are not used for pathological assessment, are usually the encouraged source of tissue for downstream research.

Despite their opportunities, only about 20 studies have been published on canine organoids/tumoroids in 2023, which is in marked contrast to the 175-fold higher output with over 3,500 studies on human organoids/tumoroids (Figure 1). Nevertheless, already 15 and 8 types of canine organoids and tumoroids, respectively, have currently been established (Table 1). Intestinal and hepatic organoids, as well as bladder and mammary tumoroids are most frequently reported (Supplementary Tables S1, S2).

Upon evaluation of the methods reported in 41 original papers, we found that all reviewed papers failed to report at least one of the required parameters as outlined in Supplementary Tables S1, S2 that could impact reproducibility and reliability of the organoid/tumoroid culturing. More specifically, the necessary parameters that were checked in the 41 original papers included dog characteristics, cell isolation specifications, cell seeding details, incubator settings, as well as used cell culture components (i.e., extracellular matrix, basal medium, activators, amino acids, antibiotics/mycotics, buffers, growth factors, hormones, inhibitors, multi-component supplements, saccharides, serum and vitamins). In addition, the clear lack of consensus regarding use of specific components in the culture media, even for similar organoid/tumoroid cultures, creates a substantial variation in protocols between research groups and could likely increase reluctance from researchers to explore these innovative animal-reducing and translatable technical tools (12–14). Our data overview is therefore also a plea to the implementation of a general standardized protocol across organoid/tumoroid types, of which the feasibility has already been demonstrated by 3 recent studies (37, 47, 49). Following comparison of the reported studies, we here suggest the potential components of such a standardized culture protocol for canine organoids and tumoroids (detailed in Table 2) based on 3 criteria, i.e., their (1) most common use, (2) lowest number of drawbacks and (3) acceptable financial impact. As starting stem cell type, ASCs and CSCs are preferred for canine organoids and tumoroids, respectively (1, 2, 74). These stem cells are most often used and are not associated with as many ethical issues as ESCs (1, 2, 74). Moreover, they can rather easy be isolated through microdissection of mostly surgery- or necropsy-derived tissue, followed by cell washing and enzymatic digestion (74, 111–114). AccuMax™ would be the preferred product to standardize the enzymatic tissue digestion as it provides a less costly, more reproducible and reliable alternative that is not mammalian- or bacterial-derived (111, 114). Matrigel^®^ is by far the preferred type of ECM in most published organoid/tumoroid culture studies and would therefore be a logical choice for protocol standardization. However, the alternative use of hydrogels should be explored in parallel to overcome the drawbacks of Matrigel^®^ (costly, animal-derived product with complex structure and variable composition) (10, 120, 121). Advanced DMEM/F-12 is the most obvious candidate for basal medium standardization as it includes multiple supplements and requires substantially less serum than other basal media (128). The preferred choice of various basal medium supplements for protocol standardization can also be clearly depicted based on their popularity in the literature, potential advantages and cost. More specifically, NAC and GlutaMAX™ should be standardized as amino acids; PS, TMS and primocin as antibiotics/-mycotics; HEPES as buffer; EGF, Noggin, Wnt3a and Rspo-1 as growth factors (and specific growth factors, such as HGF, should be added depending on the type of canine organoid or tumoroid); gastrin as hormone (and other specific hormones should be added depending on the type of canine organoid or tumoroid); A83-01 and Y-27632 as growth-supporting inhibitors; B-27 and N2 as multi-component supplements; FBS as serum supplement (although SR efforts should be encouraged); nicotinamide as vitamin derivative.

**Table 2:** Suggested standardized protocol for canine organoid and tumoroid cultures.

**Parameters**	**Cell culture component**
Cell isolation	ASCs (for organoids)/CSCs (for tumoroids), AccuMAX™
Extracellular matrix	Matrigel^®^ (use of non-animal-derived hydrogels are encouraged)
Basal medium	Advanced DMEM/F-12
Amino acids	NAC, GlutaMAX™
Antibiotics/-mycotics	PS, TMS, Primocin
Buffers	HEPES (if necessary)
Growth factors^*^	EGF, Noggin, Wnt3a, Rspo-1
Hormones^**^	Gastrin
Inhibitors^***^	A83-01, Y-27632
Multi-component supplements	B-27, N2
Serum	FBS (use of non-animal-derived SR is encouraged)
Vitamins	Nicotinamide

^*^Other specific growth factors (such as HGF) to be added depending on the canine organoid/tumoroid type. ^**^Other specific hormones to be added depending on the canine organoid/tumoroid type. ^***^Often only used in the first days of culture to boost stem cell survival and prevent anoikis.

Overall, canine organoids hold great potential in both veterinary and human medicine, but their applicability remains underinvestigated. Especially the human translation strongly relies on the hypothesis that dogs are a reliable model for humans, given their similar body size, metabolic processing, living environment, immune system, genetic diversity and disease development, including cancer (155). In fact, there are several clinical trials for drugs in dogs with cancer that provided the necessary proof-of-concept for subsequent translation and drug efficacy testing in human patients with (related) cancer. As an example, testing of the selective inhibitor of nuclear export (SINE) selinexor in dogs with lymphoma led to its accelerated FDA approval for human lymphoma patients (156–158). Similarly, evidence for the initiation of clinical trials with celecoxib as selective cyclooxygenase (COX)-2 inhibitor in human urothelial carcinoma originated from observations that COX inhibitors provide substantial reduction of urothelial carcinoma progression in dogs (159–161). One of the earliest examples of canine cancer patient trials for later human translation is the testing of toceranib against canine mast cell tumors carrying c-kit gene mutations (162). The positive results of this trial helped clinical trials with a similar tyrosine kinase inhibitor called sunitinib move forward against human cancers associated with c-kit mutations (163). Immunotherapy clinical trials are now also frequently performed in dogs as immunologically relevant models, to inform whether novel applications could also benefit humans. Testing of for example cancer vaccines against various targets is performed to demonstrate their evoked anti-tumor immune responses in dogs with human relevance (164). Moreover, transfer of adoptive T cells, chimeric antigen receptor-expressing T cells and autologous natural killer cells are explored in dogs with cancer to show homing, cytotoxicity and survival advantage of these lymphocytes and potential feasibility in humans (165). Trials in dogs have even gone beyond drug testing, and included the evaluation of medical devices and surgical techniques for human translation, with special interest for brain cancer applications through the use of canine brain tumor patients (166, 167). To the best of our knowledge, studies with canine organoids and tumoroids have not yet resulted in human applications. However, they open perspectives to quickly and reliably investigate for example druggable tumor intrinsic mechanisms with relevance for both canine and human cancer patients. Moreover, it is expected that canine organoids/tumoroids will be developed from additional tissue types by other research groups in the near future, which will further increase their translational potential and their use for initial testing of experimental drugs.

In conclusion, although the establishment of current organoids/tumoroids is quite successful, the efficiency of organoid/tumoroid generation still varies between 30 and 90% (1, 2, 5, 11, 108). This is where standardization of cell culture characteristics and rigor in methodological reporting could be beneficial. In order to become an even more important and informative preclinical tool, co-cultures of canine organoids/tumoroids with other tissue cell types (e.g., stromal and immune cells) need to be further explored (1, 2, 5, 11, 108). Moreover, currently only very few studies describe organoid vascularization, a feature that is necessary to mimic the dynamic blood flow observed *in vivo* and would also benefit the organoid life span (1, 2, 108, 168). When implemented, these additional assets will ultimately attract more scientists to canine organoids/tumoroids as tools, substantially contributing to the Cross Health concept.

## Supplementary material

Supplementary Table S1 Schematic overview of reported canine organoid parameters.

Supplementary Table S2 Schematic overview of reported canine tumoroid parameters.
